# 成人先天性支气管胆管瘘1例

**DOI:** 10.3779/j.issn.1009-3419.2010.01.18

**Published:** 2010-01-20

**Authors:** 庆伟 谭, 波 郑, 金 王

**Affiliations:** 1 116011 大连，大连医科大学附属第一医院胸外科 Department of Thoracic Cardiovascular Surgery, the First Affiliated Hospital of Dalian Medical University, Dalian 116011, China; 2 116044 大连，大连医科大学 Dalian Medical University, Dalian 116011, China

## 1 临床资料

患者，女性，51岁。咳苦胆味淡黄色泡沫样痰51年，加重2年。患者生后即出现咳嗽咯痰，夜间睡眠时加重，每日痰量约40 mL，淡黄色，味苦，最多每日达500 mL。曾于多家医院反复诊断为"肺炎"。既往无白陶土样便，无肝胆疾病史，饮食正常。查体：右侧中下胸部触觉语颤略有增强，叩诊右肺中下部可及轻度浊音，听诊右肺中下部可闻及明显湿啰音，左侧正常。支气管镜检查示：右支气管开口隆突旁可见一漏斗样狭窄分支，管腔内不断漏出胆汁样分泌物。ERCP检示：窦道经左肝管经食管裂孔越过膈肌沿食管右前方向上蔓延，查胸、上腹部CT示：可见穿过膈肌之处至纵隔造影剂进入，并见右主支气管与其分支显影。

于2009年4月9日全麻下经右胸后外侧切口行支气管胆管瘘切除结扎缝合术。术中见右下肺表面有胆汁样沉着。食管前并行一管状结构，上端与右主支气管近隆突处相连，向下经食管裂孔与左肝内相连，长约15 cm，上管口直径约1 cm，有软骨环，下管口直径约1.5 cm，肌样管状结构(图 1)。上端与右主支气管相连处根部用双7号线结扎，下端与膈肌下1 cm处双重结扎，切除瘘管，两残端再用3-0可吸收线缝扎。

**1:**
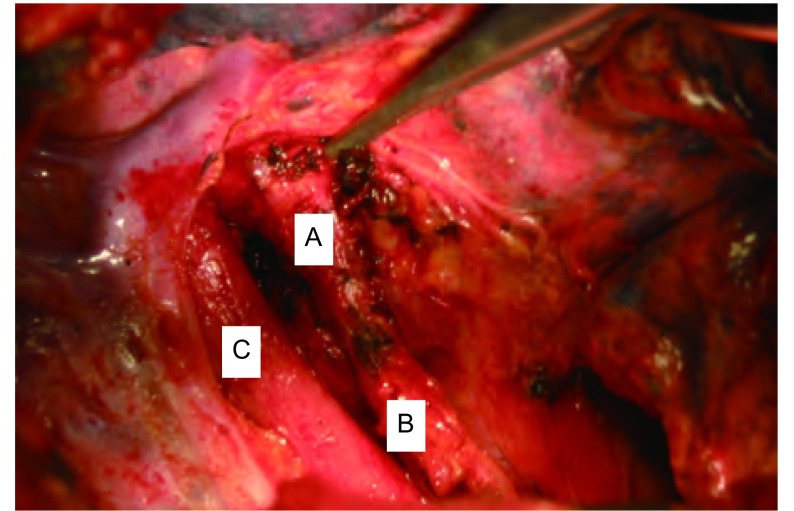
术中所见，支气管胆管瘘管 The bronchobiliary fistula in the operation

术后病理：近端见支气管性组织伴炎症改变，远端见肌样组织伴炎症改变(图 2)。诊断：1.支气管胆管瘘(先天性)；2.胆汁性肺炎。

**2:**
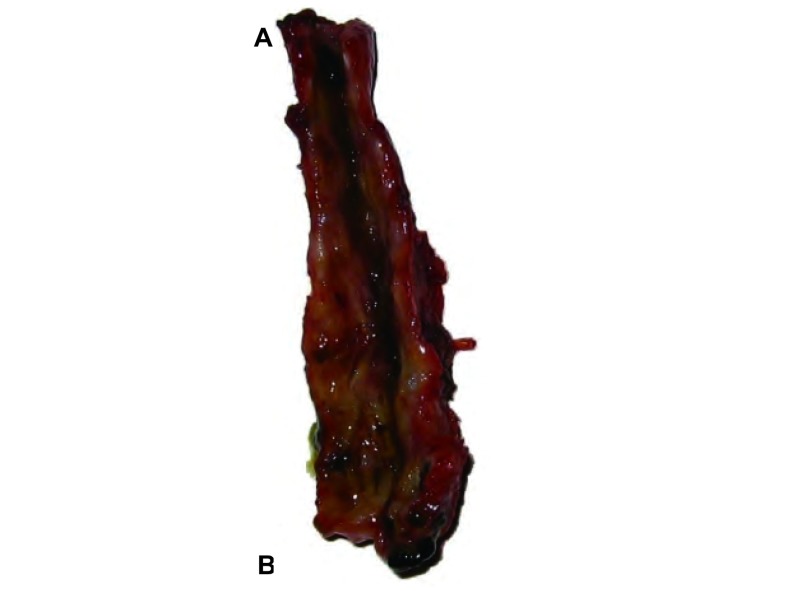
切除的支气管胆管瘘管 The excisional bronchobiliary fistula

术后患者症状消失，无并发症，痊愈出院。

## 2 讨论

先天性支气管胆管瘘是一种极其罕见的畸形，女孩较男孩常见，大多数因呼吸道症状和反复的肺部感染被发现^[1]^。成人先天性支气管胆管瘘更为罕见，文献报告仅5例^[2, 3]^。在成人病例中，胆汁流向肺组织对肺产生损伤^[3]^，但此患者没有明显的肺损伤，可能因患者能够自主地把支气管内胆汁咯出。

诊断主要依靠患者长期咳胆汁样痰及肺部感染症状病史。辅助检查包括支气管镜、ERCP或MRCP、胸上腹CT、便常规、胆汁样痰化验等。支气管镜能够很好地显示瘘管在气管内开口，并能够确定瘘管流出成分。但是ERCP或MRCP检查在确定有无胆道畸形中占有重要地位，先天性支气管胆管瘘并存胆道发育不全或胆总管闭锁者占36.8%^[4, 5]^。胸、上腹部CT检查对于继发性支气管胆管瘘有鉴别作用。

诊断明确即应手术治疗，介入栓塞疗效尚不明确。患者胆汁长期流向肺组织所致肺损伤，多伴肺部感染，术前需应用抗生素，如患者为先天性支气管胆管瘘无胆道畸形，可行右胸后外侧入路，暴露右肺和瘘管，上端尽量靠近支气管结扎瘘管或用支气管闭合器闭合，以防止残存组织继续分泌液体，对呼吸道刺激引起术后呼吸道刺激症状。下端贴近食管裂孔结扎。如患者为先天性支气管胆管瘘伴胆道畸形，开胸结扎上端瘘管同时开腹行胆道成形术。未治疗的支气管胆管瘘，可能会导致进行性呼吸功能障碍和死亡，但是如果治疗正确，预后良好，总死亡率为25%^[4]^。
